# Supplementary material for: Chromothripsis during telomere crisis is independent of NHEJ, and consistent with a replicative origin
Source: Genome Res. 2019 May;29(5):737–49. doi: 10.1101/gr.240705.118 (PMC6499312; doi:10.1101/gr.240705.118)
Supplement: Supplemental Material [file supp_gr.240705.118_Supplemental_file_1.zip › contigs/annotated_contigs/DB49/contig.2.DB49_length_462_mean_cov_11.6883116883.docx]

**DB49_length_462_mean_cov_11.6883116883**

```
AGCCTGGGGGTCCCATCACCCAGGCTTCCATACCACCCTGCACCCTCTCCATGCGCTTCCTCAGACCACAGTGTGCTGCTCCAGGAGCC
 >chr6:148608070-148608361 + E=1e-163
TATGGGTGGAGTCCAGAGGGTCAGATTTGAGGGATGAGATGTGAGTGGAGGTGCCGTTCTTGCCCCAGGTCTCTGATGGCATCAGGAGG

AGACTTCAGCTCCAGAATGCTCTCCTTCCTCCAGATGCTTGGGCAATACCCAGGACAATACCCCAAGAAGTCAGCTCCTAATGATGGCT

TCTTGAGGATCAGTAGGGCAA|TTA|GAGTGTGTGTGAGTGTGTCTGTGTCTGTGAGAGAGAAAGGGAGGAGGGAGAGAGGAAGAGAAG
                      >chr6:148874818-148874992 + E=4e-93
ATGAATGCTCATTCAGGTCTAGGTCTGGACCACTCATCAGAGAACCATGGGAACCATTATTTGGAAATATTCCAATTTCCAATACAGCA

CCCCTCCCACATGACCTCA
```
